# A Framework to Monitor Toxics: Measuring the Health Impact of Chemical Bans

**DOI:** 10.1289/ehp.117-a359a

**Published:** 2009-08

**Affiliations:** **Bob Weinhold**, MA, has covered environmental health issues for numerous outlets since 1996. He is a member of the Society of Environmental Journalists

One of the tasks mandated by the Stockholm Convention on Persistent Organic Pollutants is to determine if bans on toxic substances are effective in reducing contamination in people. But assessing such trends in diverse global populations is difficult because researchers must use changes in the average levels of contaminants measured in groups of people at different points in time—known as cross-sectional trend data—to estimate how levels are changing in individuals over time. A team of Swiss researchers has developed a pharmacokinetic model framework that may help improve the use of cross-sectional trend data in assessing the effects of chemical bans **[*EHP*** **117:1280–1286; Ritter et al.]**.

Among the parameters required by the model are the rate of elimination of the contaminant from the body and the rate of decline of individual intake of the contaminant once a ban takes effect. Additional parameters include body weight and the fraction of the body weight that is lipid. The authors tested their formulas with sample cases involving *p,p’*-DDT and *p,p’*-DDE from selected Swedish and U.K. populations, and found that the outcomes matched fairly well with concentrations identified in earlier studies.

As with any model, however, a key to its successful application is good data for the variables included in the model. The authors note that total diet studies, which are regularly conducted in a number of countries, are a good source of data to estimate changes in contaminant intake over time, because food is typically the primary source of ongoing exposures in postban situations.

The model assumes that cross-sectional averages used to estimate changes in contaminant levels over time are based on data from populations that are similar in age and other factors that would influence initial body burden and contaminant intake and elimination. The key is that changes in intakes be reasonably consistent among members of the population, but they need not be consistent for the population over time if data are available to estimate changes in dietary intake. Modeling data from populations that are reasonably similar with regard to these characteristics also means that results from the model will be population-specific and not necessarily applicable to other populations.

Still, the authors say the model is broadly adaptable, and the formulas can be modified to factor in considerations such as pathways other than ingestion, storage in body reservoirs other than fat tissue, and different elimination rates. To optimize the use of this model, the authors recommend that efforts to monitor toxics include regular data collection, including total diet data to estimate background levels of ongoing exposure, in young adult populations.

